# 2-(5-Bromo­thio­phen-2-yl)-5-[5-(10-ethyl­phenothia­zin-3-yl)thio­phen-2-yl]-1,3,4-oxadiazole

**DOI:** 10.1107/S160053681201361X

**Published:** 2012-04-13

**Authors:** Yu-Zhen Pan, You-Gui Wang, Jian-Hui Liu, Li-Cheng Sun

**Affiliations:** a College of Chemistry, Dalian University of Technology, 116024 Dalian, Liaoning, People’s Republic of China; b State Key Laboratory of Fine Chemicals, DUT-KTH Joint Education and Research Center on Molecular Devices, Dalian University of Technology, 116024 Dalian, Liaoning, People’s Republic of China; c Department of Chemistry, School of Chemical Science and Engineering, KTH Royal Institute of Technology, Stockholm 10044, Sweden

## Abstract

The mol­ecule of the title compound, C_24_H_16_BrN_3_OS_3_, contains three approximately planar fragments, *viz.* an oxadiazole ring plus two adjacent thio­phene groups, and two phenothia­zine benzene rings, with largest deviations from the least-squares planes of 0.051 (3), 0.019 (4) and 0.014 (3) Å, respectively. The phenothia­zine unit adopts a butterfly conformation, with a dihedral angle of 38.06 (15)° between the terminal benzene rings. The dihedral angle between the 2,5-bis­(thio­phen-2-yl)oxadiazole unit and the attached benzene ring is 15.35 (11)°. In the crystal, mol­ecules form stacks along the *b*-axis direction; neighboring mol­ecules within the stack are related by inversion centers, with shortest inter­centroid separations of 3.741 (2) and 3.767 (2) Å.

## Related literature

For electro-optical properties of phenothia­zine derivatives, see: Lai *et al.* (2001 ▶, 2003 ▶); Han *et al.* (2008 ▶); Meng *et al.* (2010 ▶); Zhang *et al.* (2005 ▶); Park *et al.* (2011 ▶); Kim *et al.* (2011 ▶); Hagfeldt *et al.* (2010 ▶); Wu *et al.* (2010 ▶). For related structures, see: Chu & Van der Helm (1975 ▶); Hdii *et al.* (1998 ▶); Li *et al.* (2009*a* ▶,*b* ▶); Yu *et al.* (2011 ▶); Pan *et al.* (2012 ▶).
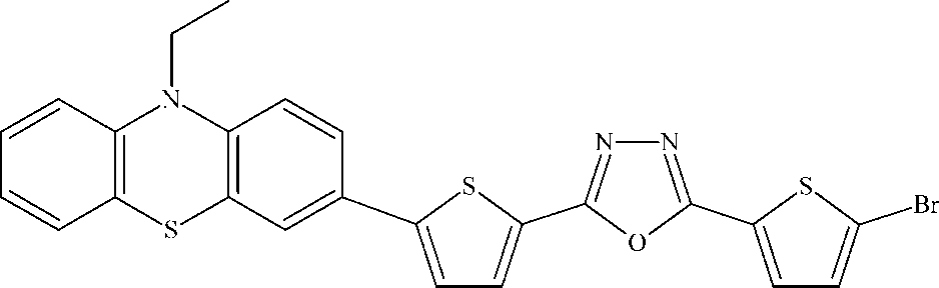


## Experimental

### 

#### Crystal data

C_24_H_16_BrN_3_OS_3_
*M* *_r_* = 538.49
Triclinic, 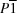

*a* = 7.4300 (5) Å
*b* = 7.6019 (5) Å
*c* = 22.1933 (14) Å
α = 89.315 (4)°
β = 89.170 (4)°
γ = 64.891 (4)°
*V* = 1134.93 (13) Å^3^
*Z* = 2
Mo *K*α radiation
μ = 2.11 mm^−1^
*T* = 296 K
0.10 × 0.08 × 0.07 mm

#### Data collection

Bruker APEXII CCD area-detector diffractometer
Absorption correction: multi-scan (*SADABS*; Bruker, 2009 ▶) *T*_min_ = 0.812, *T*_max_ = 0.860
11444 measured reflections
3972 independent reflections
3138 reflections with *I* > 2σ(*I*)
*R*_int_ = 0.052

#### Refinement

*R*[*F* ^2^ > 2σ(*F* ^2^)] = 0.044
*wR*(*F* ^2^) = 0.136
*S* = 1.06
3972 reflections
290 parameters
H-atom parameters constrained
Δρ_max_ = 0.47 e Å^−3^
Δρ_min_ = −0.62 e Å^−3^

### 

Data collection: *APEX2* (Bruker, 2009 ▶); cell refinement: *SAINT* (Bruker, 2009 ▶); data reduction: *SAINT*; program(s) used to solve structure: *SHELXTL* (Sheldrick, 2008 ▶); program(s) used to refine structure: *SHELXTL*; molecular graphics: *DIAMOND* (Brandenburg, 2004 ▶) and *SHELXTL*; software used to prepare material for publication: *SHELXTL* and local programs.

## Supplementary Material

Crystal structure: contains datablock(s) I, global. DOI: 10.1107/S160053681201361X/yk2050sup1.cif

Structure factors: contains datablock(s) I. DOI: 10.1107/S160053681201361X/yk2050Isup2.hkl

Supplementary material file. DOI: 10.1107/S160053681201361X/yk2050Isup3.cml

Additional supplementary materials:  crystallographic information; 3D view; checkCIF report

## Figures and Tables

**Fig. 1.**
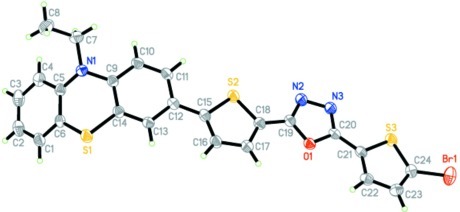
Structure of the title compound with atom-labelling scheme and displacement ellipsoids drawn at the 30% probability level.

## supplementary crystallographic information

### Comment

The derivatives of phenothiazine have attracted much interest since they can serve as photoactive and electroactive materials in many fields, such as electrogenerated chemiluminescence (Lai *et al.*, 2001, 2003), environment sensitive fluorophores (Han *et al.*, 2008; Meng *et al.*, 2010), organic light-emitting diodes (Zhang *et al.*, 2005; Park *et al.*, 2011) and solar cells (Hagfeldt *et al.*, 2010; Kim *et al.*, 2011; Wu *et al.*, 2010). As part of our studies on these materials, here we report the crystal structure of the title compound, C_24_H_16_BrN_3_OS_3_.

In the molecule of the title compound (Fig. 1), two benzene rings of the phenothiazine group display a noncoplanar butterfly conformation with a dihedral angle of 38.06 (15)°. Both thiophene rings, oxadiazole group and benzene ring with C9 atom of phenthiazine lie almost in the same plane. In the crystal, there are C—H···N contacts and π-π interactions between neighbouring molecules.

### Experimental

3-dihydroxyboryl-10-ethylphenothiazine (320 mg, 1.2 mmol), 2,5-bis(5-bromo-2-thiophen-2-yl)-1,3,4-oxadiazole (350 mg, 0.9 mmol) and Pd(PPh_3_)_4_ (21 mg, 0.02 mmol) were dissolved in 20 ml THF under nitrogen atmosphere, then aqueous solution of Na_2_CO_3_ (2.0 *M*, 6 mL) was added to reaction mixture. The mixture was stirred at 80°C for 24 hrs, then it was poured into water and extracted with dichloromethane. The organic layer was dried with anhydrous sodium sulfate. After removal of the solvent, the crude product was purified by chromatography on a silica gel column using dichloromethane-ethyl acetate (*v*/*v* = 150:1) as eluent and isolated as a yellow powder. Yield: 145 mg (30%). The yellow single crystals suitable for X-ray analysis were obtained after several days by slow evaporation of a mixture solution in dichloromethane and petroleum ether.

### Refinement

H atoms were placed in calculated positions and treated as riding atoms with C—H = 0.93–0.97 Å, and with *U*_iso_(H) = 1.2*U*_eq_(C).

### Crystal data

**Table:**

C_24_H_16_BrN_3_OS_3_	*Z* = 2
*M**_r_* = 538.49	*F*(000) = 544
Triclinic, *P*1	*D*_x_ = 1.576 Mg m^−^^3^
Hall symbol: -P 1	Mo *K*α radiation, λ = 0.71073 Å
*a* = 7.4300 (5) Å	Cell parameters from 4410 reflections
*b* = 7.6019 (5) Å	θ = 2.8–25.6°
*c* = 22.1933 (14) Å	µ = 2.11 mm^−^^1^
α = 89.315 (4)°	*T* = 296 K
β = 89.170 (4)°	Block, yellow
γ = 64.891 (4)°	0.10 × 0.08 × 0.07 mm
*V* = 1134.93 (13) Å^3^	

### Data collection

**Table:**

Bruker APEXII CCD area-detector diffractometer	3972 independent reflections
Radiation source: fine-focus sealed tube	3138 reflections with *I* > 2σ(*I*)
Graphite monochromator	*R*_int_ = 0.052
phi and ω scans	θ_max_ = 25.0°, θ_min_ = 2.8°
Absorption correction: multi-scan (*SADABS*; Bruker, 2009)	*h* = −8→8
*T*_min_ = 0.812, *T*_max_ = 0.860	*k* = −8→9
11444 measured reflections	*l* = −26→26

### Refinement

**Table:**

Refinement on *F*^2^	Primary atom site location: structure-invariant direct methods
Least-squares matrix: full	Secondary atom site location: difference Fourier map
*R*[*F*^2^ > 2σ(*F*^2^)] = 0.044	Hydrogen site location: inferred from neighbouring sites
*wR*(*F*^2^) = 0.136	H-atom parameters constrained
*S* = 1.06	*w* = 1/[σ^2^(*F*_o_^2^) + (0.0813*P*)^2^ + 0.0555*P*] where *P* = (*F*_o_^2^ + 2*F*_c_^2^)/3
3972 reflections	(Δ/σ)_max_ = 0.001
290 parameters	Δρ_max_ = 0.47 e Å^−^^3^
0 restraints	Δρ_min_ = −0.62 e Å^−^^3^

### Special details

**Table:**

Geometry. All s.u.'s (except the s.u. in the dihedral angle between two l.s. planes) are estimated using the full covariance matrix. The cell s.u.'s are taken into account individually in the estimation of s.u.'s in distances, angles and torsion angles; correlations between s.u.'s in cell parameters are only used when they are defined by crystal symmetry. An approximate (isotropic) treatment of cell s.u.'s is used for estimating s.u.'s involving l.s. planes.
Refinement. Refinement of *F*^2^ against ALL reflections. The weighted *R*-factor *wR* and goodness of fit *S* are based on *F*^2^, conventional *R*-factors *R* are based on *F*, with *F* set to zero for negative *F*^2^. The threshold expression of *F*^2^ > σ(*F*^2^) is used only for calculating *R*-factors(gt) *etc*. and is not relevant to the choice of reflections for refinement. *R*-factors based on *F*^2^ are statistically about twice as large as those based on *F*, and *R*- factors based on ALL data will be even larger.

### Fractional atomic coordinates and isotropic or equivalent isotropic displacement parameters (Å^2^)

**Table:**

	*x*	*y*	*z*	*U*_iso_*/*U*_eq_	
Br1	−0.64760 (7)	0.55973 (6)	0.18906 (2)	0.0835 (2)	
C1	0.3622 (6)	0.2273 (5)	0.96143 (15)	0.0594 (9)	
H1	0.2281	0.2762	0.9718	0.071*	
C2	0.4887 (7)	0.2710 (5)	0.99607 (17)	0.0673 (10)	
H2	0.4416	0.3463	1.0304	0.081*	
C3	0.6867 (7)	0.2023 (5)	0.97957 (17)	0.0682 (11)	
H3	0.7731	0.2313	1.0031	0.082*	
C4	0.7577 (5)	0.0905 (5)	0.92832 (16)	0.0587 (9)	
H4	0.8904	0.0489	0.9170	0.070*	
C5	0.6325 (5)	0.0397 (4)	0.89353 (14)	0.0456 (7)	
C6	0.4320 (5)	0.1113 (4)	0.91116 (13)	0.0465 (7)	
C7	0.9127 (5)	−0.2003 (6)	0.83409 (18)	0.0666 (10)	
H7A	0.9822	−0.1174	0.8320	0.080*	
H7B	0.9341	−0.2683	0.7961	0.080*	
C8	0.9994 (6)	−0.3473 (6)	0.8843 (2)	0.0839 (13)	
H8A	0.9828	−0.2808	0.9219	0.126*	
H8B	1.1383	−0.4238	0.8765	0.126*	
H8C	0.9320	−0.4307	0.8863	0.126*	
C9	0.5786 (5)	−0.0232 (4)	0.79035 (14)	0.0437 (7)	
C10	0.6538 (5)	−0.0294 (5)	0.73245 (15)	0.0522 (8)	
H10	0.7888	−0.0638	0.7269	0.063*	
C11	0.5316 (5)	0.0147 (4)	0.68302 (15)	0.0501 (8)	
H11	0.5870	0.0038	0.6446	0.060*	
C12	0.3283 (5)	0.0748 (4)	0.68915 (13)	0.0450 (7)	
C13	0.2503 (5)	0.0895 (4)	0.74764 (14)	0.0465 (7)	
H13	0.1138	0.1335	0.7532	0.056*	
C14	0.3721 (5)	0.0401 (4)	0.79706 (14)	0.0452 (7)	
C15	0.1960 (5)	0.1204 (4)	0.63696 (14)	0.0468 (7)	
C16	0.0059 (5)	0.1390 (4)	0.63392 (15)	0.0508 (8)	
H16	−0.0620	0.1199	0.6671	0.061*	
C17	−0.0786 (5)	0.1891 (4)	0.57703 (14)	0.0516 (8)	
H17	−0.2075	0.2072	0.5685	0.062*	
C18	0.0480 (5)	0.2087 (4)	0.53520 (15)	0.0488 (7)	
C19	0.0111 (5)	0.2606 (4)	0.47247 (14)	0.0469 (7)	
C20	−0.1542 (5)	0.3449 (4)	0.39177 (14)	0.0448 (7)	
C21	−0.3225 (5)	0.3959 (4)	0.35297 (14)	0.0449 (7)	
C22	−0.5041 (5)	0.4019 (4)	0.36531 (16)	0.0546 (8)	
H22	−0.5420	0.3753	0.4032	0.065*	
C23	−0.6299 (5)	0.4525 (4)	0.31502 (17)	0.0570 (8)	
H23	−0.7589	0.4620	0.3157	0.068*	
C24	−0.5401 (5)	0.4854 (4)	0.26594 (14)	0.0515 (8)	
N1	0.6991 (4)	−0.0788 (4)	0.84232 (12)	0.0492 (6)	
N2	0.1313 (4)	0.2746 (4)	0.43185 (12)	0.0563 (7)	
N3	0.0218 (4)	0.3301 (4)	0.37835 (12)	0.0554 (7)	
O1	−0.1732 (3)	0.3035 (3)	0.45107 (9)	0.0466 (5)	
S1	0.27273 (13)	0.04182 (14)	0.86988 (4)	0.0583 (3)	
S2	0.27462 (13)	0.16405 (12)	0.56662 (4)	0.0537 (2)	
S3	−0.30112 (13)	0.45375 (12)	0.27857 (4)	0.0536 (2)	

### Atomic displacement parameters (Å^2^)

**Table:**

	*U*^11^	*U*^22^	*U*^33^	*U*^12^	*U*^13^	*U*^23^
Br1	0.1064 (4)	0.0847 (3)	0.0675 (3)	−0.0479 (3)	−0.0321 (3)	0.0175 (2)
C1	0.070 (2)	0.0534 (17)	0.0436 (19)	−0.0159 (16)	0.0046 (17)	0.0046 (15)
C2	0.101 (3)	0.0543 (18)	0.046 (2)	−0.032 (2)	−0.005 (2)	−0.0026 (15)
C3	0.102 (3)	0.066 (2)	0.052 (2)	−0.051 (2)	−0.014 (2)	0.0023 (17)
C4	0.065 (2)	0.0672 (19)	0.056 (2)	−0.0394 (17)	−0.0077 (17)	0.0088 (17)
C5	0.0504 (18)	0.0507 (15)	0.0412 (17)	−0.0270 (14)	−0.0026 (14)	0.0072 (13)
C6	0.0567 (19)	0.0486 (15)	0.0362 (17)	−0.0243 (14)	−0.0032 (14)	0.0072 (13)
C7	0.047 (2)	0.078 (2)	0.068 (3)	−0.0199 (17)	0.0001 (18)	−0.0067 (19)
C8	0.071 (3)	0.077 (2)	0.083 (3)	−0.011 (2)	−0.022 (2)	0.000 (2)
C9	0.0466 (17)	0.0477 (15)	0.0412 (17)	−0.0242 (13)	0.0027 (14)	−0.0011 (13)
C10	0.0461 (18)	0.0637 (18)	0.050 (2)	−0.0267 (15)	0.0068 (15)	−0.0067 (15)
C11	0.054 (2)	0.0569 (17)	0.0405 (18)	−0.0245 (15)	0.0095 (15)	−0.0066 (14)
C12	0.0524 (19)	0.0425 (14)	0.0420 (18)	−0.0219 (13)	0.0056 (14)	−0.0029 (13)
C13	0.0435 (17)	0.0532 (16)	0.0441 (18)	−0.0219 (13)	0.0037 (14)	−0.0011 (13)
C14	0.0495 (18)	0.0504 (15)	0.0415 (17)	−0.0267 (14)	0.0020 (14)	−0.0004 (13)
C15	0.059 (2)	0.0415 (14)	0.0412 (17)	−0.0224 (14)	0.0048 (15)	−0.0023 (12)
C16	0.056 (2)	0.0576 (17)	0.0407 (18)	−0.0262 (15)	0.0045 (15)	0.0028 (14)
C17	0.0516 (19)	0.0571 (17)	0.0462 (19)	−0.0231 (15)	0.0030 (15)	−0.0027 (14)
C18	0.0534 (19)	0.0445 (14)	0.0458 (18)	−0.0184 (13)	0.0029 (15)	−0.0016 (13)
C19	0.0511 (19)	0.0452 (15)	0.0428 (18)	−0.0189 (13)	0.0016 (15)	−0.0026 (13)
C20	0.0490 (18)	0.0435 (14)	0.0403 (17)	−0.0183 (13)	0.0085 (14)	−0.0030 (12)
C21	0.0519 (18)	0.0408 (14)	0.0429 (17)	−0.0208 (13)	0.0075 (14)	−0.0007 (12)
C22	0.057 (2)	0.0539 (17)	0.052 (2)	−0.0230 (15)	0.0095 (17)	0.0028 (15)
C23	0.0512 (19)	0.0544 (17)	0.066 (2)	−0.0227 (15)	0.0007 (17)	0.0030 (16)
C24	0.063 (2)	0.0463 (15)	0.0466 (19)	−0.0247 (15)	−0.0021 (16)	0.0022 (14)
N1	0.0415 (14)	0.0610 (14)	0.0447 (16)	−0.0214 (12)	−0.0002 (12)	−0.0031 (12)
N2	0.0556 (17)	0.0717 (16)	0.0431 (16)	−0.0287 (14)	0.0030 (13)	0.0036 (13)
N3	0.0538 (17)	0.0719 (16)	0.0417 (16)	−0.0282 (13)	0.0047 (13)	0.0035 (13)
O1	0.0484 (13)	0.0497 (11)	0.0405 (12)	−0.0197 (9)	0.0060 (10)	0.0007 (9)
S1	0.0539 (5)	0.0906 (6)	0.0417 (5)	−0.0418 (4)	0.0041 (4)	0.0022 (4)
S2	0.0562 (5)	0.0677 (5)	0.0411 (5)	−0.0300 (4)	0.0038 (4)	0.0003 (4)
S3	0.0612 (5)	0.0636 (5)	0.0419 (5)	−0.0326 (4)	0.0042 (4)	0.0040 (4)

### Geometric parameters (Å, º)

**Table:**

Br1—C24	1.871 (3)	C12—C13	1.400 (4)
C1—C2	1.370 (5)	C12—C15	1.471 (4)
C1—C6	1.382 (4)	C13—C14	1.376 (4)
C1—H1	0.9300	C13—H13	0.9300
C2—C3	1.381 (6)	C14—S1	1.765 (3)
C2—H2	0.9300	C15—C16	1.361 (5)
C3—C4	1.385 (5)	C15—S2	1.736 (3)
C3—H3	0.9300	C16—C17	1.394 (5)
C4—C5	1.394 (4)	C16—H16	0.9300
C4—H4	0.9300	C17—C18	1.363 (4)
C5—C6	1.403 (4)	C17—H17	0.9300
C5—N1	1.406 (4)	C18—C19	1.441 (4)
C6—S1	1.758 (3)	C18—S2	1.726 (3)
C7—N1	1.469 (4)	C19—N2	1.295 (4)
C7—C8	1.511 (6)	C19—O1	1.358 (4)
C7—H7A	0.9700	C20—N3	1.295 (4)
C7—H7B	0.9700	C20—O1	1.369 (4)
C8—H8A	0.9600	C20—C21	1.438 (5)
C8—H8B	0.9600	C21—C22	1.355 (5)
C8—H8C	0.9600	C21—S3	1.726 (3)
C9—C10	1.387 (4)	C22—C23	1.407 (5)
C9—C14	1.406 (4)	C22—H22	0.9300
C9—N1	1.417 (4)	C23—C24	1.344 (5)
C10—C11	1.379 (5)	C23—H23	0.9300
C10—H10	0.9300	C24—S3	1.715 (4)
C11—C12	1.385 (4)	N2—N3	1.405 (4)
C11—H11	0.9300		
			
C2—C1—C6	120.6 (4)	C12—C13—H13	119.4
C2—C1—H1	119.7	C13—C14—C9	120.9 (3)
C6—C1—H1	119.7	C13—C14—S1	120.3 (2)
C1—C2—C3	119.5 (3)	C9—C14—S1	118.7 (2)
C1—C2—H2	120.2	C16—C15—C12	129.4 (3)
C3—C2—H2	120.2	C16—C15—S2	110.0 (2)
C2—C3—C4	120.6 (4)	C12—C15—S2	120.6 (2)
C2—C3—H3	119.7	C15—C16—C17	114.4 (3)
C4—C3—H3	119.7	C15—C16—H16	122.8
C3—C4—C5	120.7 (3)	C17—C16—H16	122.8
C3—C4—H4	119.6	C18—C17—C16	112.8 (3)
C5—C4—H4	119.6	C18—C17—H17	123.6
C4—C5—C6	117.6 (3)	C16—C17—H17	123.6
C4—C5—N1	122.9 (3)	C17—C18—C19	128.1 (3)
C6—C5—N1	119.5 (3)	C17—C18—S2	111.1 (3)
C1—C6—C5	120.9 (3)	C19—C18—S2	120.8 (2)
C1—C6—S1	120.4 (3)	N2—C19—O1	113.1 (3)
C5—C6—S1	118.6 (2)	N2—C19—C18	128.8 (3)
N1—C7—C8	112.8 (3)	O1—C19—C18	118.0 (3)
N1—C7—H7A	109.0	N3—C20—O1	112.7 (3)
C8—C7—H7A	109.0	N3—C20—C21	128.5 (3)
N1—C7—H7B	109.0	O1—C20—C21	118.8 (3)
C8—C7—H7B	109.0	C22—C21—C20	129.4 (3)
H7A—C7—H7B	107.8	C22—C21—S3	111.6 (3)
C7—C8—H8A	109.5	C20—C21—S3	119.0 (2)
C7—C8—H8B	109.5	C21—C22—C23	113.3 (3)
H8A—C8—H8B	109.5	C21—C22—H22	123.4
C7—C8—H8C	109.5	C23—C22—H22	123.4
H8A—C8—H8C	109.5	C24—C23—C22	111.5 (3)
H8B—C8—H8C	109.5	C24—C23—H23	124.2
C10—C9—C14	117.6 (3)	C22—C23—H23	124.2
C10—C9—N1	123.4 (3)	C23—C24—S3	113.5 (3)
C14—C9—N1	119.0 (3)	C23—C24—Br1	127.3 (3)
C11—C10—C9	121.1 (3)	S3—C24—Br1	119.19 (18)
C11—C10—H10	119.4	C5—N1—C9	117.9 (2)
C9—C10—H10	119.4	C5—N1—C7	119.3 (3)
C10—C11—C12	121.6 (3)	C9—N1—C7	118.0 (3)
C10—C11—H11	119.2	C19—N2—N3	106.0 (3)
C12—C11—H11	119.2	C20—N3—N2	106.1 (3)
C11—C12—C13	117.6 (3)	C19—O1—C20	102.1 (2)
C11—C12—C15	122.4 (3)	C6—S1—C14	98.95 (15)
C13—C12—C15	120.1 (3)	C18—S2—C15	91.68 (16)
C14—C13—C12	121.1 (3)	C24—S3—C21	90.13 (16)
C14—C13—H13	119.4		
